# Stem Cell Transplantation Therapy and Neurological Disorders: Current Status and Future Perspectives

**DOI:** 10.3390/biology11010147

**Published:** 2022-01-17

**Authors:** Mohammad Mominur Rahman, Mohammad Rezaul Islam, Mohammad Touhidul Islam, Mohammad Harun-Or-Rashid, Mahfuzul Islam, Sabirin Abdullah, Mohammad Borhan Uddin, Sumit Das, Mohammad Saidur Rahaman, Muniruddin Ahmed, Fahad A. Alhumaydhi, Talha Bin Emran, Amany Abdel-Rahman Mohamed, Mohammad Rashed Iqbal Faruque, Mayeen Uddin Khandaker, Gomaa Mostafa-Hedeab

**Affiliations:** 1 Department of Pharmacy, Faculty of Allied Health Sciences, Daffodil International University, Dhaka 1207, Bangladesh; mominur.ph@gmail.com (M.M.R.); md.rezaulislam100ds@gmail.com (M.R.I.); touhidul.ph@diu.edu.bd (M.T.I.); rashid685@diu.edu.bd (M.H.-O.-R.); mahfuzemon87@gmail.com (M.I.); mdborhan630@gmail.com (M.B.U.); sumitd00420@gmail.com (S.D.); mdsaidur569@gmail.com (M.S.R.); drmuniruddin@gmail.com (M.A.); 2 Space Science Center, Universiti Kebangsaan Malaysia, Bangi 43600, Selangor, Malaysia; rashed@ukm.edu.my; 3 Department of Medical Laboratories, College of Applied Medical Sciences, Qassim University, Buraydah 52571, Saudi Arabia; f.alhumaydhi@qu.edu.sa; 4 Department of Pharmacy, BGC Trust University Bangladesh, Chittagong 4381, Bangladesh; 5 Department of Forensic Medicine and Toxicology, Zagazig University, Zagazig 4511, Egypt; amanyrahman292@gmail.com; 6 Centre for Applied Physics and Radiation Technologies, School of Engineering and Technology, Sunway University, Bandar Sunway 47500, Selangor, Malaysia; mayeenk@sunway.edu.my; 7 Pharmacology Department & Health Sciences Research Unit, Medical College, Jouf University, Sakaka 72446, Saudi Arabia; gomaa@ju.edu.sa; 8 Pharmacology Department, Faculty of Medicine, Beni-Suef University, Beni-Suef 62521, Egypt

**Keywords:** neurodegenerative diseases, neurons, Parkinson’s disease, red blood cell, stem cell, stroke

## Abstract

**Simple Summary:**

This review highlights the scope of available treatment options for neurological diseases, especially stem cell transplantation therapy, which provides new hope. The health sector continues to grow effectively, developing new ideas for saving lives and making complex processes more accessible, such as stem cell transplantation therapy. The treatment of individual neurological disorders is associated with different pathophysiological conditions, so transplantation therapy must be performed under optimal conditions with minimal risk. The promise of stem cell transplantation increases every day, with excellent animal models and small-scale observations in human trials. Stem cell therapy provides satisfactory data that support rational therapeutic purposes.

**Abstract:**

Neurodegenerative diseases are a global health issue with inadequate therapeutic options and an inability to restore the damaged nervous system. With advances in technology, health scientists continue to identify new approaches to the treatment of neurodegenerative diseases. Lost or injured neurons and glial cells can lead to the development of several neurological diseases, including Parkinson’s disease, stroke, and multiple sclerosis. In recent years, neurons and glial cells have successfully been generated from stem cells in the laboratory utilizing cell culture technologies, fueling efforts to develop stem cell-based transplantation therapies for human patients. When a stem cell divides, each new cell has the potential to either remain a stem cell or differentiate into a germ cell with specialized characteristics, such as muscle cells, red blood cells, or brain cells. Although several obstacles remain before stem cells can be used for clinical applications, including some potential disadvantages that must be overcome, this cellular development represents a potential pathway through which patients may eventually achieve the ability to live more normal lives. In this review, we summarize the stem cell-based therapies that have been explored for various neurological disorders, discuss the potential advantages and drawbacks of these therapies, and examine future directions for this field.

## 1. Introduction

Aging is a biological phenomenon, and in humans, aging is often associated with undesirable physiological issues, including the development of neurological diseases [1]. Older nervous systems are less capable of neuronal regeneration due to the growth of inhibitory microenvironments that prevent axonal repair. Current research is aimed at the promotion of alternative cell-based approaches, including some that have been tested in limited clinical trials [2]. Neurological disorders can be categorized into three main types: diseases associated with neuronal loss from specific brain regions, such as Parkinson’s disease (PD) and multiple sclerosis (MS); diseases related to the neuronal loss subsequent to acute damage, such as stroke; and diseases associate with impaired cellular functions, such as epilepsy [3]. The etiologies that lead to the development of brain abnormalities are often multifactorial, including aging, environmental factors, chronic stress, traumatic brain injury, and gene mutations such as those identified in amyloid precursor protein (APP), presenilin-1 and -2, or apolipoprotein E (ApoE), which have all been associated with the development of neurodegenerative diseases [4].

Health researchers have profoundly investigated neurodegenerative disorders, employing various strategies to identify permanent cures [5]. One approach that has been heavily investigated is stem cell (SC) transplantation. SCs are widely believed to have significant potential for the treatment of a wide range of human diseases [6,7]. SCs are unspecialized germ cells that can differentiate into specialized cells that play specific roles in biological systems. The pluripotent properties of stem cells might be harnessed to compensate for neuronal loss or injury in the central nervous system (CNS) [8]. SC transplantation methods have become a very popular focus of recent research. A double-blind, sham-controlled surgery was performed in 2001 on PD patients and showed convincing results, encouraging researchers to progress to clinical trials [9,10].

Alzheimer’s disease (AD) is a progressive neurodegenerative disorder and the characteristics of this disorder include difficulty in performing daily tasks, confusion, and memory loss [11]. Current Alzheimer’s disease therapies are not very effective, most probably due to the huge loss of neurons in the brain. Therefore, cell-replacement therapies, including induced pluripotent stem cell (iPSC)- or human embryonic stem cell (ESC)-derived neural cells may prove beneficial in treating AD individuals, especially the patients who would not benefit from typical pharmacological treatments [12]. 

A recent study showed that the introduction of autologous hematopoietic SC (HSC) transplantation (aHSCT) treatments for patients with multiple sclerosis (MS) reduced the mortality rate from 7.3% during 1995–2000 to 1.3% during 2001–2007, with mortality rates continuing to fall to 0.7% during 2008–2016 and 0.2% during 2012–2016 [13]. MS is an immune-mediated disease [14]; unlike other standard immune-targeted drugs, aHSCT is designed to reset the immune system rather than suppress it [15,16,17]. Understanding the history of SC transplantation and the current state of SC research can provide a better perspective concerning the potential future applications of SC technologies [18]. Recent efforts have been extended to identifying methods to stimulate SC proliferation within the adult CNS and the protection of neurons and glial cells produced by endogenous stem cells. The translation of these exciting technological advances from the laboratory into clinically valuable therapies represents the next step in SC research [19,20]. 

The purpose of this review was to describe the scope of available treatment options for neurological diseases. Current treatment options are limited, and drug approval rates for new therapies remain poor compared with other therapeutic areas. SC therapy provides hope for many patients; however, this hope should be tempered by the realization that the scientific and medical communities have yet to fully unravel the complexities of SC biology and provide satisfactory data that support the rational, evidence-based application of SCs for therapeutic purposes. Few studies have progressed into extensive, pivotal investigations using randomized clinical trial designs. Obtaining results from such studies will be essential for SC therapies to gain the necessary approvals for their application as mainstream treatments in the future.

## 2. Characteristics of Various Stem Cells Utilized for Therapeutic Applications

### 2.1. Embryonic Stem Cells and Induced Pluripotent Stem Cells

**Figure 1:**
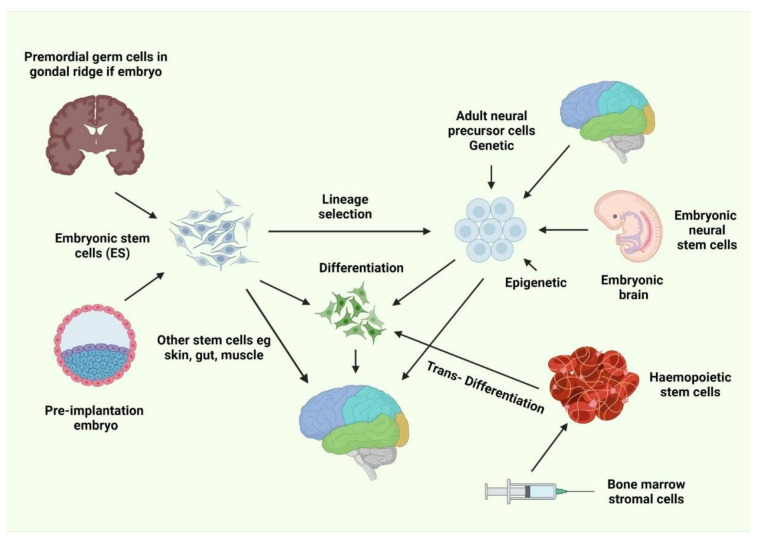
Different types of stem cells [29].

Embryonic SCs (ESCs; Figure 1) have long been utilized in numerous neurodegenerative transplantation models. In 2002, Isacson demonstrated that undifferentiated mouse ESCs could incorporate into the striatum of a rat PD model, differentiate into dopaminergic (DA) neurons, and restore the loss of motor function [21]. A few years later, two groups described the implantation of primate ESCs isolated in vitro into PD model monkeys [22] and rats [23], demonstrating the ability to integrate into the striatum, differentiate into tyrosine hydroxylase (TH)+ neurons, and partially restore motor function. Isolated ESCs can be induced to differentiate into various cells, such as oligodendrocyte precursors [24], which can relocate and differentiate into oligodendrocytes, which myelinate neuronal axons. Some ESCs can remain in an undifferentiated state [25], playing a trophic role in diminishing inflammation and maintaining the ventral myelin segments. Retinoic acid-pretreated ESCs have been shown to be effective for the treatment of rodent models of ischemia [26], in which neurological and behavioral tests indicated functional restoration. Motor neurons derived from ESCs have demonstrated the ability to alter motor functions in a rodent model of hereditary amyotrophic lateral sclerosis (ALS) [27], and multipotent neural progenitor cells (NPCs) have demonstrated the ability to reduce the clinical indications of MS in a mouse model of encephalomyelitis by reducing immune-mediated inflammation [28].

The utilization of undifferentiated ESCs has been associated with concerns about the potential to develop tumors and teratomas due to their capacity for continual proliferation. However, the induction of cell differentiation processes can diminish the multipotent ability of these cells, reducing the risks associated with transplantation; nonetheless, there remain numerous moral issues related to the use of ESCs. Other treatment avenues were introduced by Yamanaka in 2006 [30], who reported the ability to obtain early-stage undifferentiated cells from previously differentiated cells (generally fibroblasts), which have overcome the moral issues associated with the use of ESCs. These induced pluripotent SCs (iPSCs) were generated by activating four transcription factors genes: *Oct3/4*, *Sox2*, *c-Myc*, and *Klf4*. iSPCs have physiological and molecular characteristics similar to ESCs, including proliferative and differentiation capabilities. Furthermore, in vivo iPSC induction in mice has indicated that iPSCs have the incredible ability to establish developmental cell layers under empirical conditions, including the three primary germ layers (endoderm, mesoderm, and ectoderm), suggesting that the in vivo induction of iPSCs can achieve faster outcomes compared with the use of ESCs [31].

In animal models of neurological pathology, the similarities between iPSCs and ESCs make both of these cell types ideal for comparable applications. Human iPSCs have been shown to differentiate into DA precursor cells and relocate into the substantia nigra of PD rodent models, where they develop further into DA neurons over the long term and incorporate into the brain parenchyma. However, a few cells developed into tumor-like basal cells, adding to concerns regarding the safe application of these cells [32]. In another study, DA-differentiated iPSCs were isolated from a mixture of differentiated pluripotent cells using fluorescence-assisted cell sorting before implantation to decrease the risks of tumor formation [33]. Similar to ESCs, iPSCs were able to differentiate into terminal DA neurons; however, iPSCs presented more significant expression levels DA neuron-specific markers than ESCs, suggesting that iPSC-associated therapies might represent a viable avenue for the development of PD-specific treatments [34].

#### Importance of Pluripotent Stem Cells as Cell Replacements

In 2016, Takahashi and Yamanaka reported a pivotal, landmark study describing the ability to reprogram mouse fibroblast cells into a pluripotent state by activating four transcription factors OCT4 (Octamer-4), SYR (Sex Determining Region-Y, Box-2), KLF4 (Kruppel-like factor 4), and C-MYC (Cellular Myelocytomatosis Oncogenes) [30]. Since then, somatic cell lines derived from various species, including humans [35,36,37], pigs [38], mice [39], rhesus monkeys [40], marmosets [41], and sheep [42], have been successfully reprogrammed into iPSCs. Depending on the cell types, the use of fewer than four transcription factors is effective for reprogramming, and a single factor may be sufficient to obtain neural stem cells (NSCs) [43]. In addition to identifying the necessary transcription factors to derive iPSCs, various methods of transcription factor delivery have been explored. Retroviruses, lentiviruses, adenoviruses, and protein delivery methods have been used to generate iPSC lines. Temporally controlled distribution methods allow for the regulation of iPSC induction, and the use of various application sequences has facilitated the reprogramming of large numbers of cells.

Studies suggest that the expression of reprogramming factors is not required permanently, and iPSCs can efficiently differentiate into specific lineages after reprogramming; however, programmed cells (including both ESCs or other pluripotent cells) can activate endogenous pluripotency genes and silence exogenous ones. Several research groups have developed zero-footprint technologies to silence somatic cell genes permanently. Conventional techniques cannot distinguish these iPSCs from endogenous SCs. This technique has facilitated the efficient integration of Cre/Lox [44], piggyBac [45], and sleeping beauty transposons. Recently described techniques to achieve cell reprogramming include the use of specific plasmids [46] and other episomal strategies that are effectively diluted as the cells divide [47], affecting the levels of exogenous RNA [48], proteins [49], and other small molecules, reducing the probability of unintended integration events to near-zero [50].

**Figure 2:**
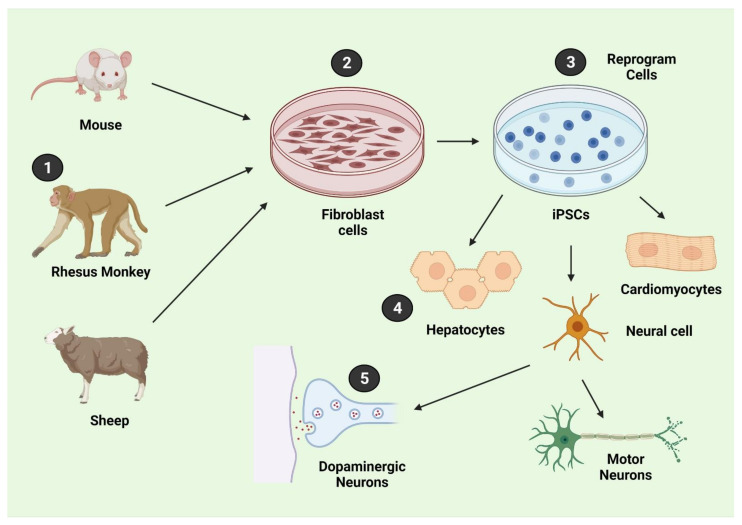
Pluripotent stem cells as cell replacements.

Human iPSCs serve as a significant source of the generation of protein-specific and disease-specific pluripotent cells [51]. The use of differentiated iPSCs for in vitro studies of neurodegenerative disease represents a promising approach for understanding the mechanisms that drive these diseases because primary human neurons are not readily available for experiments (Figure 2).

**Figure 3:**
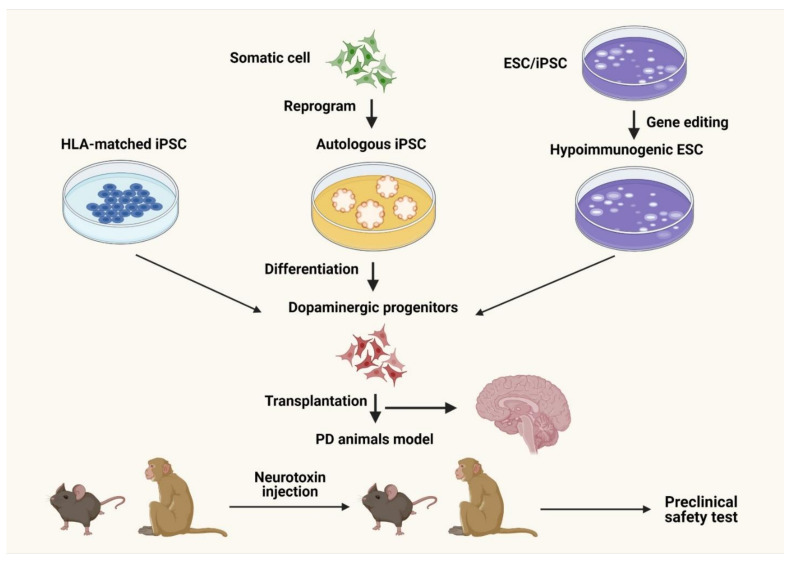
Stem cell analysis for Parkinson’s disease. Dopaminergic progenitors could be obtained from induced pluripotent stem cells (iPSCs) of various origins, including somatic cells following epigenetic reprogramming or from in vitro fertilization (IVF)-derived human embryos. Human leukocyte antigen (HLA)-matched iPSCs or gene-altered hypoimmunogenic embryonic stem cells (ESCs)/iPSCs lower the likelihood of immunogenic cell death. Preclinical tests in neurotoxin-induced PD models in fleas and nonhuman primates exhibit promising beneficial effects [53].

Cell-based analyses, including the use of MSCs, have been examined both preclinically and clinically. Many SC therapies are thought to treat disease by replacing degenerating neurons with healthy ones (Figure 3) [52,53,54].

However, the generation of research-grade autologous iPSCs is time-consuming and may require additional gene preparations to remove causative genes from patients with familial diseases. One alternative is the use of allogeneic cells to produce an HLA-matched iPSC line. To match 93% of the UK population, at least 150 homozygous HLA types from healthy donors are necessary to develop a tissue bank for the development of research-grade iPSC lines [55], which would also result in the successful matching of 90% and 41% of the Japanese and Korean populations, respectively [56,57]. This prospect would involve considerable effort, requiring the complete characterization of the various PSC lines developed. As a proof of principle, the CRISPR-mediated knockout of HLA-B in iPSCs resulted in low immunogenicity [58].

### 2.2. Neural Stem Cells

Between 9.5 and 12 weeks of gestation, the telencephalon and diencephalon of the human embryo contain cells with all the attributes of foundational SCs. They multiply at a sufficiently rapid rate to be used for the treatment of human patients with various pathologies. These cells are capable of differentiating into neurons (with physiological electrical activity), astrocytes, and oligodendrocytes [59], similar to the capabilities observed for NSCs derived from rodents [60,61].

Many animal models of neurological diseases have been effectively treated with NSCs. In a mouse model of MS, NSCs were shown to either replace damaged oligodendrocytes, allowing for the renewal of myelin sheets or the provision of trophic support through the release of various cytokines [62]. Similar cells that were administered intravenously to treat a spinal cord injury mouse model were unable to replace the damaged tissue but reduced inflammation by producing neurotrophic factors [63], suggesting that the pathologic environment can determine the outcomes for NSCs. Similar results were observed for the intraspinal transplantation of NSCs, with immunomodulatory outcomes rather than cellular replacement [64]. By contrast, the spinal infusion of human NSCs into various spinal sections of the superoxide dismutase 1 (SOD1) rodent model of ALS slowed disease progression, delayed disease onset, and increased survival rates [65].

### 2.3. Mesenchymal Stem Cells

Mesenchymal stem cells (MSCs) can be isolated from various differentiated tissues, including fat, ligaments, bone marrow stromal cells, dental pulp, skin, and fetal appendages [66,67,68]. Compared with ESCs or iPSCs, MSCs have some disadvantages; for example, MSCs present with fewer numbers, diminished proliferation capacity, and differentiation abilities with age, both in vitro and after in vivo transplantation [69]. To date, the ability of MSCs to differentiate freely has not been reported, and the one report indicating this ability, published in 2005 [70], was eventually retracted in 2010 [71].

#### 2.3.1. Bone Marrow MSCs

Various reservoirs of undifferentiated MSCs have been identified in adults. These cells were initially discovered in the bone marrow and were once referred to as “marrow stromal cells”; however, they have since been identified in various tissues, including the umbilical cord and fat tissue. In vitro, MSCs can differentiate into various tissue types and may eventually play a substantial role in regenerative medicine [72]. Bone marrow MSCS (BMSCs) can differentiate into fat, ligament, bone, and most stromal cells found in the bone marrow in vivo, playing a significant role in the protection of HSCs and regulating the hematopoietic microenvironment, deep-rooted bone regeneration, and growth [73]. Myocardial infarction (MI) results in long-lasting cardiomyocyte damage. Due to their multipotent differentiation potential, minimal immunogenicity, and excellent transferability, BMSCs may serve as suitable seed cells for the treatment of cardiovascular disease [74,75].

#### 2.3.2. Umbilical Cord Blood

In vitro studies have indicated that umbilical cord blood (UCB)-MSCs have extensive cultural memories, can be developed at a large scale, and do not develop senescence, with more significant impacts. However, UCB-MSCs display less osteogenic potential than BMSCs. The potential uses for UCB-MSCs compared with BMSCs remain under debate, and these cells have not been assessed in vivo for the purposes of bone recovery. The most widely recognized cause of paralysis in children is cerebral palsy, for which no cure currently exists. Clinical trials are currently exploring the application of UCB-MSCs to treat children with cerebral palsy-induced paralysis. Although the utilization of UCB-MSCs for neuroprotective and neuroregenerative purposes is of incredible interest, the underlying mechanisms of action that result in these effects remain poorly understood [76,77,78,79,80,81,82,83,84].

## 3. New Mechanisms of Grafted Stem Cells in Neuroregeneration

The effects of transplanted cells on the body are numerous and can be attributed to various factors, including the integration of new cells providing support to endogenous cells and immunomodulatory effects [85]. The successful incorporation of graft-derived neurons into host networks can repair damaged brain regions and restore behavioral abnormalities. The behavioral recovery observed in patients following brain-graft surgeries has been hypothesized to be due to the establishment of new synaptic connections within the brain [86,87].

In a study involving a patient with PD, researchers discovered that 24 years following transplantation, the detection of a graft-derived DA neuron-specific neuropeptide revealed the reinnervation of the putamen [88,89,90]. Glial impairments have been associated with various conditions, including stroke, MS, and Alzheimer’s disease (AD), and the replacement of these cells has been examined [91], with significant positive results (described in more detail in the section “Glial Cell Transplantation”). Grafted cells can support the survival and recovery of host neurons through the secretion of neurotrophic factors, including a brain-derived neurotrophic factor (BDNF), a nerve growth factor (NGF), and a glial cell-derived neurotrophic factor (GDNF), which are known to promote host neuron survival and promote transplanted cell survival, migration, and differentiation [92,93,94].

Another critical therapeutic mechanism involves the interaction between transplanted SCs and the immune system in the brain. Some studies have shown that SC transplantation reduces neuroinflammation, resulting in neuronal mortality [11]. In 2009, Pluchino et al. found evidence that the replacement of damaged or dead cells may not represent the fundamental mechanism underlying the apparent benefits following SC transplantation, indicating that immune modulation also plays a vital role in the observed improvements [95]. Consequently, the transplantation of SCs into the brain triggers several therapeutic mechanisms. Understanding which cell types produce positive effects, how these mechanisms are regulated, and which features may be impeding better outcomes, specifically, neuronal migration and integration into adult brain neuronal circuits, will improve the results of these therapeutic strategies [96].

## 4. Recent Advances in the Treatment of Neurological Disorder 

### 4.1. Parkinson’s Disease

The aggregation of alpha-synuclein (α-syn) is a distinctive characteristic of PD, and in vitro studies showed the promotion of fibrillation when α-syn was combined with copper, iron, or other metal ions [97]. The bimetallic nature of PD has been characterized, indicating that when serum silver, cadmium, cobalt, iron, selenium, and zinc levels decreased, aluminum, calcium, chromium, mercury, magnesium, manganese, lead, and copper were detectable [98]. Other studies have reported that patients with PD have deficient serum copper levels compared with controls [99]. Other studies have revealed a substantial increase in the iron contents of patients with PD relative to controls. High iron levels can result in vulnerability for DA cells, making them susceptible to damage. Reduced neuromelanin levels can release free iron molecules, which could cause cellular toxicity [100], and the presence of free iron increases α-syn aggregation.

PD is a chronic and progressive neurodegenerative disease that severely affects DA neurons [101]. In addition to the loss of DA neurons, the development of Lewy bodies (LBs) due to the deposition of α-syn protein aggregates in the cytoplasm of neurons is a hallmark characteristic of PD pathology [102]. In recent years, the efficacy and efficiency of non-pharmaceutical therapeutic methods for PD, including gene therapy, microRNAs (miRNAs), SC therapy, and exosomes have been studied [103,104].

The intrinsic and prospective therapeutic characteristics of the MSC secretome and MSC-condition medium, which contains the factors secreted by MSCs, can be explained by protein analysis. The therapeutic effects of the human MSC (hMSC)-derived condition medium administered to a transgenic rat model of PD include an increase in DA neurons, a partial reversal of motor impairments, and improved histopathological measures [105]. In 2017, Shin et al. discovered that the miRNA miR-17-92 clusters in MSC-derived exosomes and induces neurogenesis [106], resulting in the stimulation of oligodendrogenesis and enhanced neuronal function. Despite the limited study, the current findings show that various stem cell sources (MSCs and dental SCs) have positive benefits for PD therapy, depending on their endogenous extracellular vesicle (EV) burden.

### 4.2. Temporal Lobe Epilepsy

Medically resistant epilepsy has been associated with several negative consequences, including increased risks of accidental injury, early death, cognitive decline, and a lower quality of life [107,108,109]. Temporal lobe epilepsy (TLE) is the most common form of epilepsy and is an almost medically intractable disorder [110,111]. Most TLE patients are medically refractory, and approximately 10–20% present to epilepsy centers to obtain surgical interventions [112,113]. The aim of TLE surgical interventions is to eliminate seizures by removing the portion of the brain where seizures initiate [114,115], and a considerable proportion of TLE patients are not operative candidates. TLE patients with bilateral TLE can become permanently debilitated by the resection procedure, and even some unilateral resections result in the loss of active seizure focusing [116].

### 4.3. Multiple Sclerosis

MS is a common and disruptive disease that typically affects younger individuals [117]. MS affects individuals worldwide [118], and the underlying mechanism in disease development remains unclear. MS is a multifactorial disease associated with both genetic and environmental factors, such as vitamin D levels, ultraviolet B (UVB) exposure, Epstein–Barr infections (EBV), pollution, obesity, and smoking, increase the risks of developing MS [119]. MS is traditionally considered an autoimmune disease associated with T cell activity. However, the treatments targeted at B cells suggest that T cells may not be the only factor involved in this disease [120]. MS typically presents initially as relapsing-remitting MS (RRMS), followed by recovery or the development of secondary progressive MS (SPMS), whereas some patients present initially with primary progressive MS (PPMS), an increasingly debilitating disease [121,122]. Although the specific mechanisms underlying MS development remain unknown, various contributing factors have been identified, including EBV, UVB, smoking, vitamin D, and genetics [123,124].

Approximately six types of parenterally administered MS drugs have obtained approval from the US Food and Drug Administration (FDA), including interferons, immunosuppressants, corticosteroids, glatiramer acetate, sphingosine-1-phosphate receptor modulators, and monoclonal antibodies, which significantly reduce the frequency and intensity of MS attacks in patients with recurrent episodes by targeting the immune system at various levels through different mechanisms. N, N-Dimethyltryptamine (DMT) has been shown to reduce the frequency of relapses but shows no effects on progressive MS or axonal damage. In addition, the reported efficacy, tolerability, and safety of DMT have varied between moderate and high levels, and continued treatments have been limited by the risk of severe side effects, including cardiomyopathy [125,126].

In a mouse model of experimental autoimmune encephalomyelitis (EAE), Li investigated the influence of BMSC paracrine mechanisms, particularly the mediation of exosomes, on microglial polarization and motor function improvement [127]. Farinazzo further reported that reduced demyelination in the spinal cord following treatment with nanovesicles produced by adipose SCs resulted in decreased activity among CNS immune cells, including reduced microglial and T cell extravasation [128]. Because exosomes can penetrate the blood–brain barrier (BBB), they can deliver medicines to MS patients. The future of MS therapy is likely to be based on SC-derived exosomes for numerous reasons, including safety, the capacity to penetrate the BBB, and the ability to carry specific cargo, based on the existing literature.

### 4.4. Huntington’s Disease

Huntington’s disease (HD) is an inherited disease caused by an excessive number of CAG (cytosine-adenine-guanine) triplet repeats in the *huntingtin* gene. Copper and iron levels are higher in HD patients and model mice than in normal controls, particularly in the striatum [115], suggesting that environmental influences are unlikely to cause this dysregulation. In addition to increased copper levels, a group of copper regulatory genes has been associated with HD, and a therapeutic strategy has been proposed for HD that involves the use of a copper-binding protein [129]. Unlike copper, several movement disorders have been associated with changes in the iron levels in the brain, including PD, multiple organ atrophy, progressive supranuclear paralysis, and restless leg syndrome [130]. In addition to increased iron levels, increased manganese levels have also been reported [131].

The polyglutamine huntingtin protein, which is prone to aggregation, is transported to other cells via exosomes [132], and exosomes appear to be crucial to the development of HD pathogenesis. Exosomes have been examined for their potential to treat HD [133]. Lee et al. [134] found that exosomes from adipose-derived MSCs (ADMSCs) were able to regulate the pathogenic characteristics of an in vitro HD model by reducing intracellular mutant huntingtin aggregates and upregulating the expression of peroxisome proliferator-activated receptor-gamma coactivator 1 (PGC-1) and phospho-cAMP response element-binding protein (CREB). In addition to a decline in the intracellular expression level of RE1-silencing transcription factor (REST), the miR-124-target gene, the exosome-mediated delivery of miR-124 to the striatum of R6/2 HD transgenic mice resulted in slight improvements in behavior [135].

### 4.5. Amyotrophic Lateral Sclerosis

ALS is considered a motor neuron illness associated with the debilitating loss of muscle control. In familial ALS cases, mutations in several genes have been identified, including C9orf72, superoxide dismutase 1 (SOD1), TAR DNA binding protein (TARDBP, TDP-43), fused in sarcoma (FUS), angiogenin (ANG), alsin (ALS2), senataxin (SETX), and vesicle-associated membrane protein-associated protein B (VAPB). This phenotype is believed to be the result of a combination of genetic and environmental factors. One of the most hereditary factors associated with ALS involves the mutation of the copper–zinc binding site of SOD1 [136].

An in vitro experiment revealed that the replacement of zinc with copper binding to SOD1 increases motor neuron toxicity [136]. Furthermore, cadmium can transform SOD1 by inducing metallothionein (MT) expression, disrupting zinc homeostasis. Specifically, after MT competitively binds zinc, MT enzymatic activity increases and the activity of SOD1 reduces due to the unavailability of zinc [137]. By contrast, cadmium can impair SOD1 activity by interfering with its secondary structure, causing misfolding and, in some instances, aggregation [120]. The absence of zinc, the presence of cadmium, or high levels of copper can all contribute to ALS progression. However, serum and cerebrospinal fluid (CSF) samples from advanced ALS patients reveal enhanced zinc and copper levels [138]. Increasing iron [139] and manganese [140] levels in the CSF of patients with ALS has also been documented through the assessment of plasma L-ferritin levels, which binds iron [141]. Increased iron levels are correlated with patients’ longevity [142,143].

### 4.6. Glial and Myelin Disorders

The CNS features both neurons and non-neuronal neuroglial cells, including oligodendrocytes, astrocytes, and microglia. Neuroglial cells are typically more modest in size than neurons and are generally found in the cerebral cortex. Exploration shows that the proportion of glial cells to neurons in the male human brain is 4:1 [144], with oligodendrocytes representing the significant proportion (75.6%), followed by astrocytes (17.3%) and microglia (6.5%) [145]. Glial cells, as valuable to neurons as veins, can improve the conduction speed of neuronal transmissions by facilitating saltatory conduction along myelinated axons, allowing neurotransmissions to pass between one neuronal ganglion to another. Glia also responds to CNS damage through the initiation of gliosis, which amplifies the numbers or sizes of glial cells. Previous neuroimaging studies of posthumous brains and genome-wide association studies (GWAS) have loosely associated changes in the gray matter with the occurrence of schizophrenia [146,147,148], supported by a few converging lines of proof. Psychosis has been associated with demyelinating disorders and neurological conditions associated with myelin destruction, including metachromatic leukodystrophy, adreno leukodystrophy, cerebrotendinous xanthomatosis, Schilder’s sickness, Niemann–Pick infection, Pelizaeus–Merzbacher illness, and phenylketonuria [149,150].

### 4.7. Disorders of the Hippocampus

Hippocampal damage can negatively affect an individual’s long-term recall abilities, including difficulty creating new memories [151]. The hippocampus also plays a vital role in short-term, episodic memory, and the bilateral loss of the hippocampus ceases the formation of new memories. Although memory formation initially relies on the hippocampus, memory recovery can occur without the hippocampus [152]. Research from a lesion study assessed the need for the para hippocampal, perirhinal, and entorhinal cortices to retrieve deep-rooted memories in animals [153]. The functions of the hippocampus remain controversial, despite wide-ranging studies examining the cellular systems and behavioral effects associated with the hippocampus. Positron emission tomography (PET) has been applied by multiple functional imaging studies, which showed no hippocampal activity during the memory analysis or retrieval [154,155,156,157,158,159,160]. Therefore, memory tasks performed during functional imaging studies may not adequately challenge the hippocampus, and no substantial increases in the metabolic requirements of the hippocampus are observed during memory retrieval based on PET studies.

### 4.8. Frontotemporal Dementia

Frontotemporal dementia (FTD) is characterized by changes in personality, behavior, and language abilities and belongs to a category of rare brain disorders. After AD and dementia with LBs, FTD is the third most common form of dementia [161]. In 1892, Arnold Pick [162] first recorded FTD, which was associated with unusual apathy, a lack of empathy, and reduced self-awareness. FTD was identified by Alois Alzheimer in 1911 and is commonly referred to as Pick’s disease. People with FTD harbor irregular Pick bodies or Pick cells that are associated with disease development [163,164]. FTD is distinct from behavioral disorders, personality changes (such as primary psychiatric disease, tumors, and cerebrovascular disease), and other forms of non-degenerative dementia, especially from neurodegenerative diseases such as AD [165,166].

## 5. Stem Cell Therapy and Treatment

### 5.1. Patient Selection for HSCT in Multiple Sclerosis

aHSCT represents the most comprehensively studied comprehensive disease-modifying therapy (DMT), with limited-term toxicities. aHSCT is predominantly utilized to treat MS as an anti-inflammatory and immunomodulatory therapy, supported by considerable scientific evidence; however, aHSCT must be tolerated to be effective. Advantageous effects have been described for younger patients, including shorter illness durations, better Expanded Disability Status Scale (EDSS) scores, the reduced persistence of inflammatory disease, and the lack of other comorbidities [167,168,169,170,171,172,173,174,175,176,177,178,179]. The assessment of progress must evaluate both advances and compromises, such as the regression of stabilization of impairments or the development of new neurological conditions. aHSCT is more effective in subjects with RRMS than those with SPMS or PPMS [180].

### 5.2. Stem Cell-Based Therapy for Alzheimer’s Disease

Various SCs have been explored for therapeutic purposes, including ESCs, iPSCs, BMSCs, and ADMSCs. Neurons derived from SCs can be incorporated into existing neural networks of the host brain [181]. In animal models, SC transplantation results in increased acetylcholine levels, which enhance cognition and memory [182]. ESC-derived NPCs can differentiate into astrocytes or neurons [183]. Human iPSCs have been produced from skin cells and can differentiate into neural cells. One study indicated that iPSCs generated from the fibroblasts of a patient with familial AD could differentiate into neurons and increase the salivary levels of amyloid β (Aβ42). In addition, γ-secretase inhibitors were affected by the Aβ found in the mucus produced by the diversified neurons, suggesting that these neurons have physiologic feedback responses when treated with γ-secretase inhibitors [170].

Nearly 50 million individuals have been estimated to suffer from dementia worldwide, accounting for approximately 800 billion dollars in medical expenditures, according to estimates from the Alzheimer’s Association. The most common form of dementia is AD, which is characterized by progressive cognitive decline and the slow loss of psychological capacities. First reported in 1907 by Alois Alzheimer, AD is a multifactorial disorder, making the precise pathophysiological mechanism challenging to establish [184].

Several studies have investigated the potential for exosomes to serve as biomarkers for the early detection of AD or as a delivery vehicle for therapeutic compounds, such as nanoformulations, small interfering RNA (siRNA), and miRNAs [185]. For the early diagnosis of AD, Saman et al. utilized tau-containing exosomes generated from CSF [186]. Because Aβ and p-tau are both found in CSF-derived exosomes [187,188], CSF-derived exosomes may serve as a potentially helpful marker for the early diagnosis of AD [105]. Using combined markers (CSF p-tau and A) to diagnose AD, Clark et al. reported an 86% improvement in both sensitivity and specificity [189]. Additionally, exosome miRNA expression profile analysis can provide reasonably accurate insights into the etiology of AD patients. Several investigations have used exosomal miRNAs from various bodily fluids, including plasma and CSF, as biomarkers for AD [190].

By both direct and indirect routes, the intracerebral injection of BMSC EVs into the neocortex of the APPswe/PS1dE9 mouse model of AD reduces Aβ levels, plaque burden, and the number of dystrophic neurites in both the hippocampus and cortex. MSC EVs interact with Aβ plaques through lipid membranes, enhancing plaque phagocytosis by microglial cells to reduce plaques through the direct method. MSC EVs also contain neprilysin, a protease that degrades Aβ plaques, decreasing intracellular Aβ deposits indirectly [191]. Another study found that MSC exosomes had comparable impacts to those observed for MSCs in triggering neurogenesis and recovering cognitive functions in a rat model of AD [192].

A recent study reported by Li et al. offered information regarding the ability of NSC EVs to improve cognitive impairments in APP/PS1 AD model mice. The study’s main findings indicated that mitochondrial function-related factors, such as sirtuin 1 (SIRT1) and synaptic proteins, were overexpressed. By contrast, oxidative damage indicators, inflammatory cytokines, and microglial markers were dramatically reduced compared with the control group [193].

Another study employed exosomes generated from NSCs subjected to heat shock to treat a mouse model of AD, which successfully reversed cognitive impairments and improved motor performance [194]. Although MSC EVs have been used to treat AD in most studies, to date, two recent studies showed that EVs from different SC sources also have the therapeutic potential to improve AD-induced cognitive dysfunction via various mechanisms, such as reducing intracellular and extracellular Aβ oligomer deposition. Clinical trials of SC therapy for the management of AD are shown in Table 1.

### 5.3. Stem Cells for Treating ALS: Current Developments

ALS is a neurodegenerative disorder marked by severe motor neuron degeneration, resulting in symptoms that include muscle degeneration, weakness, fasciculation, and spasticity [203]. ALS is the world’s most prevalent motor neuron degenerative disease, with a national incidence and frequency of 2–3 per 100,000 and 4–66 per 100,000 [204], respectively, resulting in a high burden for both stakeholders and society. Patients tend to die within 3 to 5 years of disease onset due to gradual motor neuron loss and skeletal muscle weakness, especially the muscles responsible for respiration, leading to ALS-induced mortality [205]. A promising alternative cure for ALS is SC therapy, given the exceptional plasticity and ability of SCs to differentiate into various neuronal lineages [206]. Consequently, SCs represent a valuable source of cellular replacement. When transplanted locally or systemically, stem cells can migrate to disease-related loci to exert therapeutic effects [207]. Several stem cells can be used to alter the disease pathophysiology in modern cell therapies [208], including slowing or halting disease progression, likely by supplying surrounding cells with defensive factors [209,210,211,212,213,214].

### 5.4. Encapsulation of hPSC-Derived Pancreatic Progeny for Cell Therapy

The creation of pancreatic beta cells from human pluripotent stem cells (hPSCs) represents a promising cell replacement therapy for diabetes. The status of the actin cytoskeleton and the pancreatic transcription factors that drive pancreatic origins were established in this study. According to both bulk and single-cell RNA sequencing, various degrees of actin polymerization biased cells against endodermal differentiation pathways, and neurogenic 3-induced endocrine differentiation was substantially limited by circumstances favoring a polymerized cytoskeleton. Latrunculin A was applied to depolymerize the cytoskeleton during endocrine induction, resulting in a spatial differentiation technique to produce hPSC-derived cells with increased in vitro and in vivo mobility. SCs were trained to secrete insulin in response to glucose signaling after being separated from four hPSC lines. The transplantation of islet-sized cell aggregates successfully and rapidly corrected severe pre-existing diabetes in mice. Treated mice maintained normoglycemia for at least nine months, similar to the rate observed for human islets. hPSCs represent a powerful tool for treating illnesses at the cellular level [30,215]. The differentiation of pancreatic progenitors or pancreatic beta cells derived from hPSCs requires an acceptable transplant site and a suitable encapsulation material or system. The pancreas offers an acceptable microenvironment for islet maturation, but the means for delivering and retrieving these hPSCs remains limited [216].

### 5.5. Immune Inflection and Suicide Gene Approach for Improving the Safety of Beta Cell Therapy

Diabetes mellitus occurs due to the failure or dysfunction of insulin-secreting beta cells in the pancreas. Diabetes can be classified as type 1 or type 2. Type 1 diabetes is determined by the loss of beta cells, whereas type 2 diabetes involves the development of insulin resistance, characterized by beta-cell dysfunction, in response to a mixture of hereditary and environmental factors [216]. hPSCs are a potent tool for cell therapy in both forms of diabetes.

Two types of hPSCs can be utilized: embryonic stem cells (hESCs), which are obtained from the inner mass of a developing embryo, and induced hPSCs (hiPSCs), derived through the reprogramming of somatic cells. In vitro, hPSCs can be engineered to include a final initiation point for the differentiation of beta cells and can be transformed into any cell type using the proper signaling molecules [30,215]. However, stepwise differentiation protocols were designed to direct cells toward the differentiation of pancreatic progenitor cells to generate mono-hormonal insulin-secreting cells within the body [217]. Although great advancements in encapsulation technology have been made, challenges remain when attempting to engraft transplanted cells. The elimination of the encapsulation system would expose the graft to immune system degradation. hPSCs, however, offer a powerful genome-editing tool that allows for the deletion of human leukocyte antigens (HLAs) that induce an immune response, allowing for the production of universal hPSC donor lines.

Interestingly, although pancreatic progenitors derived from hPSCs exhibit low levels of HLAs, HLA expression was upregulated in beta cells during in vivo maturation [218]. Islet cells demonstrate heterogeneity during development, which may indicate the presence of multiple populations of pancreatic progenitors. Due to islet architecture and beta-cell plasticity changes, heterogeneity may also develop postnatally [219].

### 5.6. Strategies to Enhance Cell Survival after Transplantation: Genetic Modification and Hypoxic Preconditioning

The survival of the transplanted cells will significantly increase the therapeutic efficacy of transplantations. Therefore, designing a plan for the primary outcome in the treatment of damaged tissues can assist in the avoidance of apoptotic cell death [220]. The ischemic myocardium must be placed in a hostile environment for transplanted cells to survive. Various treatment methods have been proposed to address the risks associated with apoptotic cell death. Exposure to transient hypoxia or anoxia can pre-condition cells, resulting in less-than-desirable effects and rendering them vulnerable to subsequent lethal ischemic injury [221]. Therapeutic transgenic delivery to the heart, either through direct injection or the engraftment of the genetically engineered donor SCs expressing the transgene-encoding vector, has yielded promising outcomes [222,223,224]. In tandem with cell transplantation, the transmission of growth factor genes to the heart has produced encouraging results by supporting donor cell survival [225]. A vital initiator of the apoptotic cascade is the lack of matrix conformity. For intramyocardial transmission, the suspension of donor cells in fibrin glue can significantly increase their retention in the infarcted heart, and fibrin also improves cell graft survival by supplying an unstable extracellular matrix to support the transplanted cells following intramyocardial injection [226]. Alternatively, fibrin glue injections into the ischemic myocardium can induce neovascularization, improving geographic blood flow and providing improvements in cell oxygenation. Similarly, collagen, which is a standard extracellular matrix component, has been shown to promote cell survival and growth both in vitro and in vivo, following transplantation into ischemic hearts, contributing to the improvement of left ventricle contractile function [227].

### 5.7. Transplanted Neural Stem Cell Therapy for Brain Ischemic Stroke

Ischemic stroke represents a common cause of death and injury, with no available treatments. SC transplantation represents a potential therapeutic avenue, as stroke causes irreversible neuron damage and neural tissue injury. NSCs are unique SCs that only form in the CNS and can differentiate into neurons, astrocytes, and oligodendrocytes, which can compensate for deficiencies in endogenous neurons and enhance cell survival in the inflammatory microenvironment.

Globally, stroke represents one of the top three causes of death and injury and can be classified into two types: ischemic stroke and hemorrhagic stroke, which accounts for more than three-quarters of all stroke events (approximately 80–85%) [228]. However, the treatment of ischemic stroke must be individualized and includes heterogeneous therapies, many of which are closely associated with the location of the ischemic injury, patient age, and the capacity for neuronal self-repair. The critical goal of therapeutic stroke care is the restoration of regional cerebral blood perfusion as rapidly as possible following stroke diagnosis to prevent the incidence and degree of impaired dysfunction [229,230]. Exogenous NSC transplantation therapies are still far from acceptable for clinical applications due to myriad legal, therapeutic efficacy, and safety issues that remain to be resolved. Currently, few human trials have been completed, although several preclinical animal experiments have been performed [231,232,233,234,235]. Many preclinical animal studies of ischemic stroke have examined the therapeutic effectiveness and protective capabilities of transplanted exogenous NSCs. Their findings have revealed that exogenous NSCs can substantially enhance the prognosis of animal models of ischemic stroke; not only were clinical outcomes improved, but the histological assessment of the infarcted area showed a significant decrease, without apparent safety concerns. Two critical pathways have been identified to describe the treatment effects of exogenous NSCs for ischemic stroke [236,237,238,239,240].

miRNAs are involved in cellular and molecular processes, including cellular senescence, telomere length, and circadian rhythms, and can effectively be transferred into SCs. EVs harvested from cells targeted by miRNAs often contain an abundance of miRNAs, which can be applied to the treatment of age-related diseases, including stroke and AD [241]. Exosomes extracted from angiotensin-converting enzyme 2-expressing human placental MSCs improved post-stroke outcomes in an acute ischemic stroke model, according to Barzegar et al. (2020). In addition to neurological recovery, these exosomes displayed protective effects against the negative consequences of ischemic stroke [242].

Similarly, a preclinical study in animals found that nano-sized EVs derived from BMSCs encouraged neurological recovery by decreasing leukocyte infiltration in the brain, resulting in ischemic neuroprotection and reducing neurological deficits [243]. MSC EVs demonstrated therapeutic potential in a rat stroke model examined by Moon et al. They discovered that exosomal cargo, such as miRNA-184 and miRNA-210, mediated the effects of MSC EVs in the induction of neurogenesis and angiogenesis [244].

In in vitro and in vivo models of ischemic stroke, Sun et al. investigated the anti-ischemic effects of exosomes derived from two types of SCs, NSCs and hiPSC-derived cardiomyocytes. In vitro ischemic damage was induced by oxygen-glucose deprivation (OGD) in primary mouse astrocytic or neuronal cells, followed by exosomal treatment. In OGD-exposed astrocytes, NSC-derived exosomes provided substantial protection [245]. Overall, these findings demonstrate that SC-derived exosomes obtained from a cell-free therapeutic approach could be used to treat stroke-related damage. An analysis of cell transplantation for treating both ischemic and hemorrhage stroke models is shown in Table 2.

### 5.8. Stem Cell Transplantation in Stroke Clinical Trials

Individual experiments exploring the efficacy of SC transplantations in the treatment of stroke are ongoing. Recent research confirmed that human neuronal cells successfully engrafted onto the stroke-damaged brain region survive for up to 2 years after the initial engraftment in a single patient [266]. Other cell transplantation studies in patients with PD have noted the survival of engrafted cells for up to 14 years after transplantation [267].

SC transplantation requires additional research prior to translation into real-world clinical applications. The development of SC-based paradigms for stroke treatments requires that experts, clinicians, managers, and industry delegates establish guidelines through preclinical and clinical assessments. A set of basic guidelines for SC-based research were established in 2009 [268], followed by an update in early 2011 [269]. An analysis of existing stem cells research for stroke treatment enhances the likelihood that a therapeutic strategy will emerge for clinical translation [270]. The clinical trials in case of stroke by using stem cell therapy is shown in Table 3.

### 5.9. Extracellular Vesicles Derived from Mesenchymal Stem Cells Protect against Neonatal Stroke by Interacting with Microglial Cells

MSC EVs are derived from BMSCs and can be characterized according to their size distribution (NanoSightTM), and their MSC origins and localization were confirmed by identifying protein markers. The damaged and contralateral cortices of postnatal day 9 (P9) mice were extracted and cultured after a 3-h transient middle cerebral artery occlusion (tMCAO). MSC EV treatment reduced the injury volume 72 h after tMCAO, in part via modulatory effects on microglial cells. MSC EVs were primarily detected in Iba1^+^ cells and GLUT1^+^ blood vessels in the ischemic-reperfusion area after 72 h [296].

## 6. Neuro-protective Role of SCs in Neuroinflammation

Neurological problems disturb the brain’s and spinal cord’s normal function and are a leading cause of mortality and disability globally. Speech, memory, sensorimotor, and autonomic functions are all affected by central nervous system dysfunction, which can have a significant impact on a patient’s quality of life. The neuro-protective role of SCs in neuroinflammation are shown in Table 4.

The success of cell transplantation and its efficacy to treat neuroinflammation is determined by several parameters, including the route, dosage, and time of administration, but the cell type used is the most essential [305].

Importantly, stem cell therapy offers a treatment paradigm that is especially suited to combating both acute and chronic inflammatory conditions. Researchers have long emphasized the need for neuroprotection during the subacute period of stroke and other bran injuries because inflammation often occurs during this period and, if left untreated, can greatly aggravate the extent of injury [306]. Both the subacute and chronic stages of neuroinflammation require neuro-regeneration and the maintenance of anti-inflammatory activities [307,308]. Chronic stem cell therapy is intended to activate brain rejuvenation and reperfusion by stimulating regenerative mechanisms such as vasculogenesis, neurogenesis, angiogenesis, and synaptogenesis [309]. It can restore cerebral infrastructure, such as the BBB, and sequester inflammatory insults, such as oxidative stress and mitochondrial impairment [310,311].

Stem cell therapy has the potential to address an alarmingly gloomy vacuum in known subacute and chronic treatments for neuroinflammatory patients by supporting the damaged brain in healing from an ischemic or hemorrhagic event by moderating endogenous neuroinflammation [312] and stimulating reinnervation [313]. Fetal cells, NT2N cells, CTX0E3, embryonic stem cells, neural stem/progenitor cells, umbilical cord blood, amnion, adipose, and induced pluripotent stem cells have been investigated in laboratory experiments over the years [314,315,316,317,318].

While several of these cell types have been studied in clinical trials for ischemic stroke and other neuro disorders such as AD, PK, and ALS (amyotrophic lateral sclerosis), much of the current preclinical research and clinical trials have focused on bone marrow cellular derivatives [319]. Other disease indications have shown that bone marrow-derived stem cells, such as mesenchymal stem cells (MSCs), endothelial progenitor cells (EPCs), SB623, multipotent adult progenitor cells (MAPCs), and multilineage-differentiating stress enduring (Muse) cells, have a good safety profile [320]. Furthermore, bone marrow-derived stem cells, particularly MSCs, have been widely examined in animal models.

Transplanted NSCs reduce neuroinflammation, increase neurogenesis, and restore cognitive performance in Alzheimer’s disease animal models [321]. In addition, NSC implantation reduced cross-communication between NSCs and endothelial cells. As a result, NSC-based therapy for Alzheimer’s disease could provide an optimal neural microenvironment to prevent neurodegeneration and ensure the survival of mature neurons [322].

MSC transplantation into AD models showed neuroprotective potential via modifying neuroinflammation, promoting endogenous hippocampus neurogenesis, reducing neuro apoptosis, and enhancing the signaling pathway. Transplantation of bone marrow-derived MSCs (BM-MSCs) into mouse AD models, for example, reduces neuroinflammation and improves neuropathology and cognition [323,324].

A study conducted by Neelam K. Venkataramana and colleagues [325] indicated that stem cells have a beneficial effect. Seven PD patients, aged 22 to 62 years old and with a mean disease duration of 14.7 7.56 years, participated in a prospective, uncontrolled pilot research of single-dose, unilateral autologous bone marrow-derived mesenchymal stem cell transplantation (BM-MSCs). After 36 months of follow-up, three of the seven patients demonstrated a significant improvement in their Unified Parkinson’s Disease Rating Scale (UPDRS) score of 38 percent [326].

Stem cell therapy may be able to help people with ALS live longer. This is accomplished by stem cells’ ability to specialize in specific supporting cells like astrocytes and microglia (cells within the central nervous system). These supporting cells may halt the degeneration of motor neurons in the central nervous system [327].

In animal models of neuroinflammation, laboratory evidence evaluating whether the same stem cell population is capable of both preventative/protective and restorative actions [328,329]. However, because most inflammatory episodes are unpredictable, using stem cells as a preventive or protective treatment in the clinic may be limited, suggesting that stem cell therapy is better suited as a regenerative biologic.

## 7. Discussion

### 7.1. Ethical Issues and Safety Concerns Regarding hESC-Based Therapies

Human embryonic stem cells (hESCs) originate from the pluripotent inner cell mass of pre-implantation embryos’ [330,331]. Octamer-limiting record factor 3/4 (OCT3/4), stage-unequivocal lacking antigens 3 and 4 (SSEA-3 and SSEA-4), TRA-1-60, TRA-1-81, and acid neutralizer phosphatase represent standard pluripotent SC markers, associated with enhanced levels of telomerase development and normal karyotypes. Under in vitro and in vivo conditions [332,333], hESCs can develop into any of the three germ layers (endoderm, mesoderm, and ectoderm) and any cell type. With improved cell substitution techniques, hESCs hold the remarkable promise of the eventual treatment of human disease [334].

### 7.2. Surgical Safety Aspects of Cell Transplantation

The damaged spinal cord in patients with ALS can tolerate sequential microinjections delivered to the cervical and thoracolumbar spinal regions. Patients with either transcendent bulbar ALS or ongoing immunosuppression might be vulnerable to increased periprocedural risks. Regular preclinical examinations are expected to create, additionally, (1) the capacity to determine the necessity for immunosuppressant use, (2) imaging modalities equipped to distinguish post-implantation engraftment, limitations, and efficacy, and (3) improved methods for identifying allogeneic human engraftments in the spinal cord of human recipients. We have recently obtained an FDA endorsement to advance into a stage II preliminary trial, which will determine toxicity limits and determine the endurance limits of the ALS spinal cord.

Further, this study will evaluate the scope of modifiable treatment parameters that can be endured (e.g., infusion number, infusion sites, complete portion conveyed). When characterized, the resulting multicohort preliminary studies will evaluate the optimal procedure and the feasibility of this treatment approach. The ALS spinal cord may serve as an ideal setting to ascertain the resilience of the spinal cord while determining the optimal method for delivering SC-based therapeutics and evaluating the signs and symptoms that indicate successful engraftment. Long-term goals will focus on the clinical approval of a focused microinjection approach for the application of SC-based therapies to a wide range of spinal cord afflictions [335].

### 7.3. Optimizing the Therapeutic Efficacy of Neural Stem Cell Transplantation

Although preclinical studies have confirmed the efficacy and safety of NSC transplantations for treating ischemic stroke, a few points of contention remain. The engraftment and survival rate of NSCs in vivo is less than 5%, indicating that various issues should be addressed before this methodology can be translated into clinical applications [336,337,338,339]. The critical issues that must be optimized include in vivo competence and NSC differentiation following transplantation. Both endogenous and exogenous NSCs are more likely to differentiate into glial cells than neurons in vivo [340,341,342]. Different studies have attempted to adjust the NSC contents at the protein level through many methods, such as viral transfection, heat pre-treatment, antibody treatments, and cytokine treatments to determine whether any of these effects can shift differentiation toward neurons [343].

## 8. Future Perspectives

The health sector continues to grow effectively, developing new ideas for saving lives and making complex processes such as SC transplantation therapy, which plays a significant role in drug development and bioscience research and is likely to improve very effectively [344]. The numerous cell types and their origins that can be used in cell therapies for neurological diseases are depicted schematically in this diagram [18]. To be effective, SC therapy for neurological diseases must fulfill specific conditions. First, the grafted cell should differentiate into the cell type of interest, both in vivo and in vitro. Second, the grafted cells must integrate into the local neural network. Third, the half-life of the grafted cell should be extended. Fourth, and most importantly, tumor formation should not occur. Last, the pathological microenvironment of an animal model might affect the safety and efficacy of SC therapy [345]. The majority of neural autoimmune diseases are likely to benefit from the development of aHSCT. ADMSCs may cause a potent immune-suppressive effect [346]. Human ESCs can be obtained from the inner cell mass of blastocysts, and fetal brain cells can be obtained from aborted fetuses. iPSCs can be obtained by reprogramming modified cells, such as human fibroblasts, and MSCs can be obtained from cord blood or bone marrow. These unique cell types can be developed into neuronal prototypes and implanted into damaged brains [347]. Moreover, transplantation with ESCs might produce a tolerant immune system that crosses HLA barriers [348]. Some critical factors that may affect the therapeutic outcomes of SC transplantation include the SC type, the administration route, the administration dose, and mechanisms of activity, all of which should be analyzed in future clinical trials [349,350,351]. For the future development of SC transplantation therapies, the most crucial step is to design and perform trials at experienced centers with experience in both transplantation and autoimmune diseases, and utilize new strategies and international collaborations to facilitate timely comparisons with current best standards of care in the context of well-designed, randomized clinical trials [352].

## 9. Conclusions

Based on extensive previous research, the underlying mechanisms of action that lead to the development of neurological diseases remain under investigation. The promise of SC transplantation increases daily, with excellent results reported in animal models. However, human trials must still be performed, although some small-scale experiments have been conducted, which have revealed the potential for severe side effects. The treatment of individual neurological disorders will be associated with different pathophysiological conditions; therefore, transplantation therapy must be performed under optimal conditions with minimal risk. Before performing transplantations in humans, basic research remains essential [344]. As described by Cao et al., current research offers no strong evidence or certainty that the SC process can do what it is intended to do, and it has not yet reached its expected level [353]. Determining the exact mechanisms that guide NSC differentiation can be problematic in the context of CNS injury, but choosing these pathways may be crucial to the future of successful SC-based treatment strategies for patients [353].

## Figures and Tables

**Table 1:** Clinical trials of stem cell therapy for the management of Alzheimer’s disease.

Intervention Model	Route of Administration	Cell Source	Disease Condition	Number of Patients	Clinical Trial Phase	Primary Outcome	Clinical Trial Identifier	References
Single group assignment	Intravenous	Human umbilical cord blood-derived mesenchymal stem cell (MSC)	Dementia of the Alzheimer’s disease (AD) type	9	Phase I	Number of participants with adverse event	NCT01297218	[195]
Single group assignment	Intravenous	Human umbilical cord blood-derived MSC	AD	30	Phase Ⅰ/Ⅱ	Number of participants with adverse event	NCT01547689	[195]
Crossover assignment	Subcutaneous	Filgrastim	AD	8	Phase I/II	Change in ADAS-Cog and Selected CANTABS tests	NCT01617577	[196]
Case-Control	Brain surgery	Human umbilical cord blood-derived MSC	AD, Dementia, Brain diseases, Central nervous system diseases, Nervous system diseases, Tauopathies, Neurodegenerative Diseases, Delirium, Dementia, Amnestic, Cognitive Disorders, Mental disorders	14	Phase I	Incidence rate of adverse events	NCT01696591	[197]
Parallel assignment	Intraventricular	Human umbilical cord blood-derived MSC	AD	45	Phase I/II	Number of subjects with adverse events	NCT02054208	[198]
Parallel assignment	Peripheral intravenous	Longeveron MSC	AD	33	Phase I	Incidence of treatment-emergent serious adverse events	NCT02600130	[199]
Parallel assignment	Intravenous	Human umbilical cord blood-derived MSC	AD	16	Phase I/II	Change in ADAS-Cog score	NCT02672306	[200]
Parallel assignment	Intravenous	Human umbilical cord blood-derived MSC	AD	45	Phase I/II	Change from the baseline in ADAS-Cog	NCT03172117	[201]
Parallel assignment	Peripheral intravenous	Longeveron allogeneic human MSC	AD	33	Phase I	Incidence of treatment-emergent serious adverse events	NCT02600130	[199]
Parallel assignment	Peripheral intravenous	Autologous bone marrow-derived stem cells	AD, Vascular dementia, Lewy body disease, Lewy body dementia with behavioral disturbance, Mixed dementia, Parkinson-dementia syndrome, Chronic traumatic encephalopathy, Huntington’s dementia, Wernicke Korsakoff syndrome, Traumatic brain injury, Multi-Infarct dementia, Autism, Autism spectrum disorder Autistic behavior, Autistic disorder, Cadasil, LATE limbic-predominant age-related TDP-43 encephalopathy	100	Not Applicable	Running	NCT03724136	[202]

**Table 2:** Cell transplantation medical care analysis in ischemic and hemorrhage stroke models [246].

Administration Route	Initiation Time Point	Cell Type/Dose	Species/Model	Outcome	Mechanism	References
Intracerebral	One month	NT2N line/0.8 m	Rat/tMCAO	Motor purpose retrieval	Biobridge, cell standby, persuaded evolution, and trophic provision	[247,248]
	Seven days	hBMSC	Rat/tMCAO	Sensorimotor salvage	Tempted progress and trophic backing	[249,250]
	Fourteen days	MHP36 line/0.2 m/8 μL	Rat/tMCAO	Sensorimotor recapture	Cubicle additional	[251,252]
	Seven days	hBMSC	Rat/ICH	Sensorimotor repossession	Made increase and trophic issues	[253]
	Seven days	hNSC/	Mouse/ICH	Motor role regaining	Cell spare	[254]
	Seven days	hNSC/0.8 m/2 μL	Rat/Endothelin	Motor role regaining	Cell auxiliary	[255]
	Seven days	hES/0.2 m/4 μL	Stroke Mouse/Barrel Stroke	Sensorimotor recuperation	Cell additional	[256]
	Seven days	rESC/0.1 m	Rat/MCAO	Endurance and diversity of implants	Cell renewal	[257]
Intracranial	Seven days	miPS/0.4 m/4 μL	Rat/Barrel Stroke	Sensorimotor retaking	Cell replacing	[258]
Intravenous		rBMSC/1 m/1 mL	Rat/tMCAO	Dipping alteration, motor recouping	Hinder endothelial disfunction	[259]
	Twenty-four hours	hUCBC	Rat/tMCAO	Sensorimotor replevin	Cell substitution	[260]
	Twenty-four hours	rMSC/3 m	Rat/tMCAO	Sensorimotor reclamation	Red-reducing cell death	[261]
	-	hNSC	Rat	Sensorimotor recovery	Cell replacement	[262]
		hNSC/5 m/500 μL	Rat/ICH			
Intra-arterial	One hour	rBMSC/1 m/1 mL	Rat/tMCAO	Relying reducing infarction	Induced growth and trophic item	[263]
	Twenty-four hours	hBMSC/1000		Sensorimotor recapture	Decreasing swelling	[264]
Intranasal	Six hours	rBMSC/1 m/100 μL	Rat/Barrel Stroke	Dipping infarction, sensorimotor recovery, better-quality olfactive roles, and neuropsychiatric aids	Prompt germination and trophic factors	[265]

**Table 3:** Stroke clinical trials using stem cell therapy [246].

Trails	Initiation Year and Country	Cell Source and Administration Route	Population	Outcome	Status	References
Safety	-	NT2/D1 and Intracerebral	Basal ganglia stroke	Feasible	Completed	[266,271]
	2001, USA	NT2/D1 and Intracerebral	Stroke patients	Feasible with small risk of seizure	Completed	[272]
	-	MSC and Intravenous	MCA	-	-	[273]
	2005, USA	ES and Intracerebral	Ischemic stroke patients	2/5 patients showed improvements	Terminated	[274]
	2008, India	BMMNC and Intrathecal	Stroke patients	-	Completed	[275]
	2009, Cuba	BMSC and Intracerebral	Stroke patients	Good tolerance and safety	Completed	[276]
	2010, Brazil	BMMNC and Intra-arterial	Nonacute ischemic stroke	Feasible and safe	Completed	[277]
	2011, Japan	MSC and Intravenous	Stroke patients	Feasible and safe	Completed	[278]
	2012, Hong Kong	UCBMC and Intracranial	Stroke in the middle cerebral artery territory and stable hemiplegia or hemiparesis	N/A	Completed	[279]
	2012, Brazil	BMMNC and Intra-arterial	MCA acute ischemic stroke	Safe	Completed	[280]
	2010, UK	NSC and Intracranial	Stroke patients		Ongoing	[231]
	2011, Taiwan	OEC and Intracerebral	Thromboembolic Stroke	-	-	[281]
	2012, China	HSC and Intra-arterial	Internal carotid artery territory infarction	N/A	Recruiting	[282]
	2014, Spain	BMMNC/2m/kg or 5m/kg and Intra-arterial	Moderate-to-severe acute ischemic stroke patients	Appears to be safe; 30% clinical improvement at 90 days	Recruiting	[283]
	2014, China	NSC and Intracerebral	Chronic ischemia stroke	-	Completed	[284]
	2014, China	EPC and Intravenous	Chronic ischemia stroke	-	Recruiting	[285]
	2015, China	UCMSC/20m and Intravenous	ICH	-	Ongoing	[286]
	2016, China	UCMSC and Intravenous	Intracerebral ischemic stroke	-	-	[287]
	2016, Taiwan	ADSC and Intracerebral	Stroke patients	-	-	NCT02813512 [288]
Efficacy	2008, Japan	BMMNC/25 mL and Intravenous	Stroke patients	-	Completed	[289]
	2009, Taiwan	CD34+ Stem Cell and Intracerebral	Chronic stroke adult patient	-	Completed	[290]
	2011, USA	BMSC/2.5m 5.0m or 10m and Intracranial	Chronic stroke patients	No serious adverse events attributable and significant improvements in motor impairment	Completed	[291]
	2014, UK	NSC and Intracerebral	Stroke patient Phase II	Strongly positive results for 12 months; Well-tolerated/no serious adverse events	Ongoing	[292]
	2014, India	BMMNC and Intravenous	Ischemic stroke Phase II	Safe but no beneficial effect	Recruiting	[293]
	2016, US/UK	Multi stem cells and Intravenous	Ischemic stroke Phase II	Excellent; 12-month functional improvement	Completed	[294]
	2016, Europe	ADSC/1m/kg and Intravenous	Hemispheric ischemic stroke	-	Recruiting	[281]
Effectiveness	2013, China	MSC and Intrathecal	Cerebral palsy	-	Recruiting	[295]

This list is not an exhaustive collection of all ongoing clinical trials, but it includes a sample of available studies from published papers, searchable websites, and ClinicalTrials.gov Identifiers.

**Table 4:** Significant neuroinflammatory mediators [297].

	Family	Types	Produced By	Role	References
Cytokines	Pleiotropic polypeptides (glycoproteins)	Tumor necrosis factor-α (TNF-α), IL-1β, IL-6, IL-20, IL-10, and transforming growth factor (TGF)-β	Microglia	Neuroinflammation (TNF-α, IL-1β, IL-6, IL-20)	[298,299]
			Astrocytes	Neuroprotection (IL-10 and TGF-β)	
			Neurons and Endothelial cells		
			Invading leukocytes		
Chemokines	Small cytokines (classified into subgroups according to variations in cysteine residues)	Monocyte chemoattractant protein 1 (MCP-1), macrophage inflammatory protein-1α (MIP-1α), and fractalkine	Microglia	Pro-inflammatory as chemoattractants for invading leukocytes	[300]
			Astrocytes		
			Injured neurons		
Cellular adhesion molecules (CAMs)	Cell surface proteins (often transmembrane receptors)	Immunoglobulin superfamily (IgSF), integrins, cadherins, selectins	Endothelial cells	Pro-inflammatory by facilitating extravasation of invading leukocytes	[301]
			Epithelial cells		
			Leukocytes		
Reactive oxygen species	Free oxygen radicals	Superoxide anion radical (O_2_•^−^), singlet oxygen (^1^O_2_), hydroxyl radical (·OH) and perhydroxyl radical (HO_2_·), nitric oxide (NO)	Neuronal, endothelial* and inducible NO synthases (n−, e−, iNOS, respectively), Oxidative imbalance	Ischemic cell death	[302]
				Endothelial NO production can have a neuroprotective effect	
Matrix metalloproteases	Zinc-containing endopeptidases	MMP-2 (gelatinase A) and MMP-9 (gelatinase-B)	Endothelial cells	Pro-inflammatory via degradation of BBB to facilitate invasion of peripheral leukocytes	[303]
			Neutrophils		
			Macrophages		
Regulatory T cells	Lymphocytes	CD4^+^CD25^+^	Dendritic or antigen-presenting cell	Immunosuppressive	[304]
				Mediate microglial/astrocytic activation	

## Data Availability

Not Applicable.
